# Exploring how members of the public access and use health research and information: a scoping review

**DOI:** 10.1186/s12889-023-16918-8

**Published:** 2023-11-07

**Authors:** Celayne Heaton-Shrestha, Kristin Hanson, Sophia Quirke-McFarlane, Nancy Delaney, Tushna Vandrevala, Lindsay Bearne

**Affiliations:** 1 Kingston University, Faculty of Health, Science, Social Care and Education, Kingston-upon-Thames, KT27LB UK; 2 University of Surrey, Department of Psychology, Guildford, GU2 7XH UK; 3 Guy’s and St Thomas’ NHS Foundation Trust, Department of Physiotherapy, London, SE1 7EH UK; 4 St George’s, University of London, Population Health Research Institute, 1st Floor Jenner Wing, Cranmer Terrace, London, SW17 0RE UK

**Keywords:** Health research, Health information, Public, Patients, Access, Use, Scoping review

## Abstract

**Background:**

Making high-quality health and care information available to members of the general public is crucial to support populations with self-care and improve health outcomes. While attention has been paid to how the public accesses and uses health information generally (including personal records, commercial product information or reviews on healthcare practitioners and organisations) and how practitioners and policy-makers access health research evidence, no overview exists of the way that the public accesses and uses high quality health and care information.

**Purpose:**

This scoping review aimed to map research evidence on how the public accesses and uses a specific type of health information, namely health research and information that does not include personal, product and organisational information.

**Methods:**

Electronic database searches [CINAHL Plus, MEDLINE, PsycInfo, Social Sciences Full Text, Web of Science and SCOPUS] for English language studies of any research design published between 2010–2022 on the public’s access and use of health research or information (as defined above). Data extraction and analysis was informed by the Joanna Briggs Institute protocol for scoping reviews, and reported in accordance with the PRISMA extension for scoping reviews.

**Results:**

The search identified 4410 records. Following screening of 234 full text studies, 130 studies were included. One-hundred-and-twenty-nine studies reported on the public’s sources of health-research or information; 56 reported the reasons for accessing health research or information and 14 reported on the use of this research and information. The scoping exercise identified a substantial literature on the broader concept of ‘health information’ but a lack of reporting of the general public’s access to and use of health research. It found that ‘traditional’ sources of information are still relevant alongside newer sources; knowledge of barriers to accessing information focused on personal barriers and on independent searching, while less attention had been paid to barriers to access through other people and settings, people’s lived experiences, and the cultural knowledge required.

**Conclusions:**

The review identified areas where future primary and secondary research would enhance current understanding of how the public accesses and utilises health research or information, and contribute to emerging areas of research.

**Supplementary Information:**

The online version contains supplementary material available at 10.1186/s12889-023-16918-8.

## Background

Making high-quality health and care information available to members of the general public is crucial to support populations with self-care and improve health outcomes, as knowledge ‘holds the potential to change practice and achieve positive clinical, population and other outcomes,**’** [1] (p.524). Minimally, ‘high quality information’ may be understood as information grounded in primary research, free from commercial sponsorship and other conflicts of interest [2]. Additional criteria such as conciseness, simplicity of design, and continued updating may be required by some authorities for research-based information to be considered ‘high quality information’ (e.g. [3]).

The science of how people access and use health information is not new (e.g. [4]). However, if the requirement of ‘high quality’ for health information is adopted, that is, that the information be ‘research’ or ‘research-based’, the existing literature presents a number of shortcomings. Firstly, the literature that has examined how research is accessed and used has tended to focus on practitioners and policymakers (e.g. in the emerging field of Research on Research use [5]), with relatively little attention paid to how members of the public access and use research. Secondly, while a rich literature exists on how the public access and use health information, it has tended to conflate all types of health information – including research evidence and information such as personal records, medication labels and physician’s personal web pages [6]. Consequently, little is known about how the public accesses and uses high quality health information, and there are no summaries or overviews of this topic.

In this light, a scoping review methodology was deemed appropriate as such reviews are intended to ‘map the literature and provide an overview of evidence, concepts, or studies in a particular field’ and the results may be used to inform priorities for future research on the topic of interest [7].

Accordingly, this review aimed to systematically search for and describe the research evidence on how members of the public access and use (high quality) health research or information (HRI) relating to human health and healthcare; the reasons for access and use of HRI and the factors that may shape how they access and use HRI. In order to approximate the notion of ‘high quality information’, the review adopted a narrower definition of ‘health information’ than in the broader literature, excluding personal records, product information, and information on establishments providing healthcare.

## Methods

The review was informed by the Joanna Briggs Institute guidance for conducting scoping reviews and reported in accordance with the Preferred Reporting Items for Systematic reviews and Meta-Analyses (PRISMA) Extension for Scoping Reviews [8, 9]. The search was conducted in three steps: an initial search of a select number of academic databases (CINAHL plus, MEDLINE and Web of Science) to identify and narrow the range of relevant search terms to inform the final search strategy; an expanded search of academic databases (CINAHL Plus, MEDLINE, PsycInfo, Social Sciences Full Text, Web of Science and SCOPUS) with the identified search terms; and manual search of the reference lists of included systematic reviews and meta-analyses. Alongside, experts in the field were consulted to ensure all relevant studies had been included in the retrieved corpus.

This search strategy departed from the current JBI guidance on scoping reviews as neither grey literature nor manual searching of the reference lists of all included studies was conducted, due to resource constraints.

The protocol was registered with the Open Science Forum (registration https://doi.org/10.17605/OSF.IO/RXP39) on 16/02/2022.

### Data sources

Search terms included subject headings, free text and wild-card terms located in the title or abstract for population of interest (members of the public e.g. general public, public, people, community, lay public, lay person, patient, carer), concept of interest (access to and use of human health research or information. e.g.: access*, utilisation/utilisation, us*, adopt*, uptake, engagement; AND research evidence, research findings, research publications, research articles, research outputs, scientific evidence, scientific findings, scientific articles, scientific publications, scientific knowledge, research, information) and context of interest (e.g. health, healthcare).

The search was limited to studies published between 01–01-2010 and 18–01-2022. This was informed by the rapid changes in communications technologies over the last decade and evidence that most studies on the use in healthcare of social media, a technology able to reach less traditional audiences [10], were published after 2010 [11] (Table 1). The full electronic search strategy is presented as Supplement 1.

**Table 1:** Inclusion and exclusion criteria

Inclusion criteria	Exclusion criteria
- Studies investigating access to and use of health and health care research or information (as defined in this study) by members of the public	- Studies that discuss or report access to research or information relating to topics other than health and healthcare - Studies in which ‘health information’ includes personal records, personal, product or institutional information only or as well as health research evidence, and data on each type of information is not presented separately - Studies that focus exclusively on health care professionals and students/trainees - Studies that focus on non-human health (e.g. animal, planetary)
- Participants from any socio-cultural background, age, gender, ability and profession	
- Any research design	- Studies not written in the English language
- Study dated to from 1^st^ January 2010	- Studies published prior to 2010
- Published, peer-reviewed, full-text articles	- Opinion pieces, editorials, protocols, conference abstracts and proceedings, commentaries, books and book chapters, unpublished dissertations, evaluation reports

### Study selection

Studies were eligible for inclusion in this review if: they investigated the access and use of HRI by members of the general public from any socio-cultural background, age, gender and ability, and national setting, following any research design, and they were published in the English language in peer-reviewed journals. The inclusion of English language only publications was due to the limited availability of resources for translation.

Access to HRI was defined as the process of finding and obtaining HRI or physically accessing HRI in varied formats. Studies which discussed how information is accessed conceptually only (e.g. National Institute of Health and Care Research (NIHR) [12]) were not included. HRI use or utilization was defined as what people did with the research or information they had accessed, including how they assessed, applied or adapted the research or information to their needs and context [13] rather than their intention or stated preference. Studies which discussed ‘access to health information’ where it was clear that by ‘health information’ was meant personal health records, information about physicians, hospitals or medication labelling or similar types of information (personal, product and institutional information) only were not included. Studies in which ‘health information’ included these last types of information as well as research evidence and data for each was presented separately, were included.

### Collating, summarising and reporting the results

Records were exported to Proquest® RefWorks for deduplication and then exported to Rayyan (Rayyan https://www.rayyan.ai/). Independent (blind) screening of abstract/titles against eligibility criteria was completed by two reviewers [CHS, KH]. The two reviewers initially screened 25 records independently and then conferred to establish common understanding. Each reviewer screened 50% of remaining records and then checked 20% each other’s screening for accuracy. One reviewer [CHS] screened all full-texts against the eligibility criteria, and a second reviewer [KH] checked 5%. Any disagreements were resolved through discussion. A third reviewer was identified as arbitrator, though this was not needed [LB or TV].

A bespoke data extraction tool was developed and piloted on five included studies (See Additional file 1). Two reviewers [SQM, CHS] extracted data from included studies, and a third reviewer [ND] checked 10% of the extracted data for accuracy.

Data were extracted on: study characteristics (author/s, date, title, journal, keywords, study type, methodology); population characteristics; reasons/purpose for accessing/using HRI (general interest, specific condition); source of HRI; utilization of accessed HRI; condition/aspect of health or healthcare to which the HRI accessed relates; and factors facilitating access or barriers to accessing the HRI. Data for each category was summarised in table form, accompanied by a narrative.

**Fig. 1:**
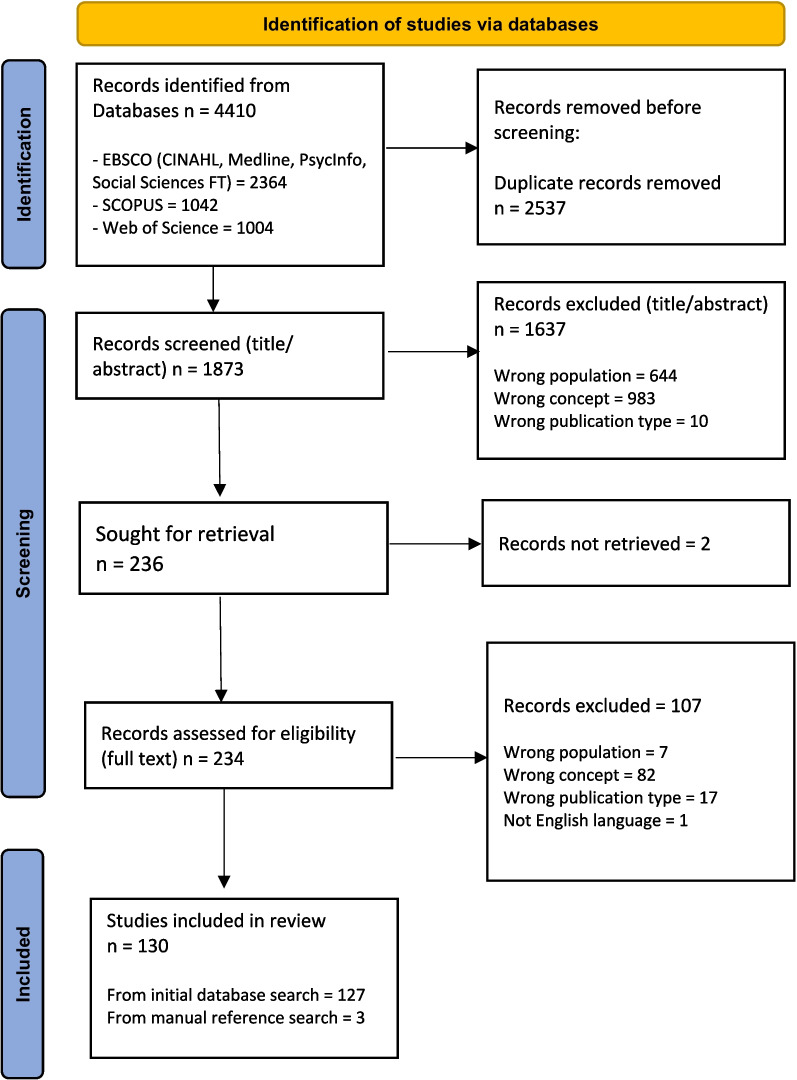
PRISMAScR diagram

Figure 1 presents a flow diagram for the scoping review process adapted according to the PRISMA extension for scoping reviews (PRISMA-ScR) statement [14].

## Results

### Study characteristics

The search produced 4410 records. Following deduplication and title and abstract screening the full text of 234 studies were screened and 130 studies were included in this review (Fig. 1).

Two studies investigated access to research by members of the public [15, 16]. One hundred and twenty-eight studies investigated access to health information by members of the public (Supplement 2).

Eighty included studies (62%) applied a quantitative research methodology [17–93], 33 studies (25%) followed a qualitative methodology [15, 94–125], 13 studies (10%) were mixed- or multi-method studies [16, 126–137], and four (3%) were reviews [138–141].

Fifty-nine included studies were conducted in North America (45%) [15, 17, 30, 33–35, 38, 39, 42, 43, 46, 49–51, 54, 56, 60–62, 66, 67, 69, 71, 75, 76, 78, 79, 82–84, 87, 88, 91, 94–96, 98–105, 107, 108, 114–116, 118–122, 124, 128, 129, 136, 137], 18 in Europe (14%) [16, 24, 26, 27, 53, 57–59, 74, 77, 85, 86, 111, 113, 117, 125, 127, 142], 18 in Asia (14%) [19, 28, 29, 48, 55, 65, 68, 73, 80, 81, 89, 90, 97, 110, 123, 132, 134, 135], 11 in Africa (8%) [20, 25, 31, 45, 52, 63, 64, 72, 112, 126, 130], nine in the Middle East (7%) [18, 22, 23, 32, 47, 93, 109, 143, 144], five in Australasia (4%) [40, 41, 44, 70, 133] and two in South America (2%) [37, 106]. Four studies spanned several continents (3%) [21, 92, 131, 139] and another four studies did not state any specific geographical location (3%) [36, 138, 140, 141].

The studies included people with specific health conditions (*n*=33) [21, 25–27, 29, 31, 35, 45, 51–53, 66, 69, 74, 78, 84, 86, 90, 94, 97, 99, 100, 118, 125, 129, 131], hearing or visual impairment (*n*=4) [22, 107, 119, 133], carers (*n*= 11) [18, 23, 37, 50, 51, 91, 99, 104, 109, 131, 132], the elderly (*n*=6) [44, 67, 72, 85, 87, 134], youth or teens (*n*=12) [32, 35, 64, 67, 82, 94, 119, 129, 130, 135, 137, 140], minority populations (*n*=22) (e.g. ethnic minorities [33, 38, 39, 42, 61, 75, 96, 98, 101, 105, 114–116, 118, 122, 139], homeless people [60, 62] or refugees [41, 46, 88, 111], and criminalised individuals [102]. Twenty-four studies included other populations (e.g. African American breast cancer survivors [95], members of public libraries [143], women in Tanzania [126] a rural community [127], students in an ESOL class [17, 28, 34, 41, 47, 60, 62, 67, 70, 80, 83, 93, 95, 106, 110, 112, 113, 117, 120, 123, 124, 126, 127, 145]. Eighteen studies were a sample of the general population [16, 19, 24, 43, 48, 49, 56, 58, 68, 73, 77, 79, 81, 92, 108, 121, 128, 144] and sixteen studies did not identify the population [15, 20, 21, 30, 36, 71, 76, 89, 98, 103, 116, 118, 136, 138, 140, 141]. Some study populations had several of the characteristics listed above.

### Access to health research and information by members of the public

Sixty-one studies listed healthcare professionals (including GPs, nurses, allied health professionals, complementary and alternative therapists) as a source of HRI. Sixty studies mentioned informal sources (friends, work colleagues, families and neighbours); and 18 studies mentioned other types of professional advisors, such as pastors, educators, governmental officials or charity sector workers (Table 2).

**Table 2:** Sources of HRI for the general public

Source	No. of studies	Study number
**Other people as source of HRI**		
Healthcare professionals (*n* = 61)		
Unspecified healthcare professionals	36	[15, 18–20, 26, 28, 32, 34, 37, 42, 43, 45, 47, 49, 52, 57, 58, 62, 65, 75, 85–88, 98, 102, 111, 112, 116, 117, 119, 120, 124, 138, 140, 144]
Doctors	33	[23, 25, 27, 33, 37, 42, 51, 53, 57, 58, 69, 72, 75, 77, 81, 88, 90, 93, 98, 101, 104, 108, 112, 113, 118, 123, 126–128, 133, 135, 142, 144]
Allied Health Professionals	16	[23, 25, 38, 58, 62, 81, 82, 101, 104, 108, 111, 112, 118, 138, 142, 144]
Nurses	8	[23, 25, 45, 81, 101, 112, 118, 144]
Alternative medical practitioners	5	[38, 72, 112, 118, 123, 126]
Informal sources (*n* = 60)		
Family, friends, and/or colleagues	52	[16, 19, 20, 22, 25–28, 31, 32, 37, 38, 43, 45, 49, 53, 58, 62, 69, 70, 72, 75, 77, 81, 82, 85, 88, 98, 101, 102, 106–109, 111–114, 119, 120, 122, 123, 126–128, 130, 133, 135, 138, 140, 144, 145]
Peers/people experiencing similar condition	8	[24, 25, 43, 45, 57, 86, 104, 109]
Unspecified	5	[34, 92, 111, 116, 117]
Other professional advisor (*n* = 18)		
Individuals identified as scientists or having access to scientific knowledge	2	[16, 104]
Religious practitioners	1	[123]
Formal education figures	4	[32, 82, 104, 119]
Government officials (including public health)	4	[16, 31, 72, 77]
Non-government organizations/Charities	4	[31, 62, 72, 126]
Other sources	8	[16, 22, 34, 52, 88, 111, 138, 142]
**Specific settings as source of HRI**		
Medical (*n* = 14)		
Primary care	9	[25, 26, 77, 84, 86, 100, 114, 116, 126]
Secondary care	8	[31, 38, 42, 73, 77, 84, 98, 114]
Other	1	[31]
Community (*n* = 20)		
Town hall meetings	1	[20]
Community meetings/health centres	5	[52, 77, 98, 116, 123]
Age group meetings	1	[72]
Churches/Religious Gatherings	8	[20, 25, 31, 52, 109, 112, 114, 116]
Support groups	9	[33, 95, 98–100, 103, 113, 131, 144]
Formal education (*n* = 5)		
Secondary education	1	[130]
Tertiary education	2	[82, 140]
Unspecified education setting	3	[114, 133, 140]
Other training settings (*n* = 14)		
Conferences/Seminars/Lectures/Workshops, etc	14	[19, 20, 26, 31, 32, 47, 58, 62, 86, 108, 111, 112, 120, 134]
Other (*n* = 12)		
Libraries/Book shops	12	[20, 25, 32, 45, 51, 62, 88, 113, 117, 121, 124, 143]
**Tools used in independent searches for HRI (n = 83)**		
Social media (*n* = 27)		
Social media (unspecified)	18	[16, 18–22, 24, 32, 45, 47, 59, 65, 81, 87, 90, 125, 139, 143]
Facebook	5	[21, 55, 84, 101, 139]
Twitter	3	[21, 55, 139]
Reddit	1	[21]
YouTube	6	[36, 55, 76, 101, 132, 139]
WhatsApp	2	[55, 59]
Instagram	1	[55]
Pinterest	1	[55]
WeChat	2	[55, 89]
MySpace	1	[139]
Telegram channel	2	[55, 109]
Search engine (*n* = 19)		
Search engine (not specified)	11	[19, 21, 24, 47, 59, 89, 90, 99, 100, 120, 125]
Google	7	[19, 55, 70, 84, 101, 122, 128]
Yahoo	2	[122, 145]
Naver	1	[122]
Database (unspecified)	1	[121]
Websites (*n* = 25)		
Health/disease/condition-specific websites	15	[19, 24, 28, 45, 47, 55, 62, 71, 89, 90, 93, 99–101, 106]
Healthcare providers/service-related websites (physician, hospital, pharmacy, etc.)	4	[24, 47, 125, 142]
Personal websites	2	[65, 93]
Health insurance websites	2	[86, 142]
Pharmaceutical websites	1	[142]
Government websites	4	[22, 99, 100, 132]
Online Encyclopaedias	2	[89, 125]
Web portal	3	[85, 89, 142]
Other unspecified websites	3	[52, 81, 93, 109]
Online Communities (*n* = 13)		
Online discussion forum	9	[19, 47, 57, 84, 89, 99, 100, 144, 145]
Internet communities	1	[142]
Chat rooms	1	[125]
Online Q&A board/Chat reference service	3	[89, 97, 121]
Scholarly/Academic sources (*n* = 16)		
Medical/Health/Scientific/Academic Journals and/or magazines	13	[19, 20, 23, 27, 46, 69, 99, 113, 117, 121, 128, 136, 145]
Textbooks/Medical Encyclopaedias	3	[15, 98, 117]
Periodicals	1	[122]
Mass media (*n* = 51)		
TV (satellite, cable, etc.)	37	[15, 16, 19, 20, 22, 23, 25–28, 32, 34, 37, 45–47, 52, 58, 62, 70, 72, 75, 81, 84, 87, 98, 101, 107, 108, 113, 114, 124, 127, 128, 138, 144, 145]
Radio	26	[15, 16, 19, 20, 23, 26, 27, 32, 34, 37, 45, 46, 52, 58, 62, 70, 72, 75, 81, 84, 87, 108, 112, 115, 138, 144]
Newspapers and/or magazines (print, online)	33	[16, 19, 20, 22, 25–28, 34, 37, 38, 42, 46, 52, 58, 62, 65, 70, 72, 73, 75, 84, 87, 88, 108, 122, 126–128, 138, 144]
Other mass media (unspecified)	6	[31, 49, 73, 86, 130, 140]
Phone services and applications (*n* = 13)		
Landlines	2	[40, 41]
Telephone services	1	[44]
Health help telephone lines	1	[84]
Telephone (with whom not specified)	1	[121]
Telephone information number	1	[88]
Over the phone (type of phone and with whom not specified)	1	[92]
Unsolicited text messages	1	[84]
Electronic devices and applications	9	[24, 32, 40, 41, 59, 86, 89, 120, 126]
Various printed informational materials (*n* = 48)		
Poster	7	[18, 23, 25, 26, 31, 45, 142]
Pamphlets/Leaflets/Brochures	21	[19, 23, 25, 34, 42, 45, 53, 58, 77, 86, 88, 98, 108, 114, 121, 124, 126, 127, 134, 136, 138]
Books	27	[16, 19, 20, 26, 27, 37, 38, 42, 58, 62, 73, 75, 77, 86–88, 113, 116, 117, 120, 121, 126, 127, 136, 138, 144, 145]
Print media/materials (type not specified)	6	[49, 51, 81, 84, 119, 122]
Written (e.g. notices to health examination, test results)	1	[111]
Newsletters	1	[65]
Paper based guidelines/materials	3	[92, 114, 117]
Marketing materials (*n* = 3)		
Campaign	1	[18]
Commercial marketing	1	[132]
Medical bill board	1	[45]
Other online sources (*n* = 2)		
Online sources (not specified)	1	[72]
Web-based health info	1	[85]
Other sources (*n* = 10)		
Local materials and resources (not specified)	1	[95]
Podcast	1	[126]
Films	1	[126]
Non-science resources	1	[104]
Video services	1	[59]
Favourites lists (not specified)	1	[144]
Worksites	1	[114]
Video instructions	1	[134]
Music, dance, drama	1	[84]
Formal education assessments	1	[82]
Postal	1	[121]

Forty-five studies listed a type of setting (a place or event) as the source of HRI, including medical settings (*n* = 14), formal community settings such as town hall meetings (*n* = 20), formal educational settings (*n* = 5), other educational settings (*n* = 14) such as workshops/lectures, and settings such as bookshops or libraries (*n* = 12) (Table 2).

Finally, 83 studies reported on the tools used by members of the public to access HRI. This comprised: mass media (*n* = 51), printed information (*n* = 48) the internet (*n* = 38). Internet sources included social media (*n* = 27); various specialist governmental, non-governmental and personal websites (*n* = 25); and search engines (*n* = 19). Online communities of various types (platform unspecified) were mentioned as a way to access HRI in 13 studies. Other sources mentioned among included studies were scholarly sources such as academic journals, textbooks and encyclopaedias (*n* = 16), phone services and applications (*n* = 13), and marketing materials (*n* = 3) (Table 2).

### Reasons for accessing and using health research and information

Fifty-six studies reported on reasons for seeking HRI by members of the public. The main reasons for seeking HRI were: (i) to find health-related information for other people and on different topics (*n* = 46); (ii) to navigate the healthcare system, such as preparing for meetings with healthcare professionals (HCPs) and advocating on one’s behalf, making one’s own health decisions, including whether to seek professional help, and sometimes to avoid going to an HCP, and to verify, clarify or add to information received from other sources; to manage one’s own health (*n* = 31); and (iii) to obtain psycho-social support by reading testimonials from other people, gain reassurance and comfort, and to gain a sense of control over the diagnosis, condition or treatment (*n* = 9) (Table 3).

**Table 3:** Reasons for seeking or accessing health research or information (HRI)

	Number of studies	Study number
To look for health information for:		
Oneself	4	[17, 44, 50, 93]
Someone else	11	[17, 18, 44, 50, 55, 58, 91, 93, 99, 115, 117]
To look for health-related information on the following topics (*n *= 46):		
General health information	11	[19, 23, 39, 44, 48, 55, 58, 72, 83, 93, 128]
A specific disease / condition including its - symptoms - diagnosis - prognosis - transmission - causes - complications - other/unspecified	36	[17–20, 24, 25, 27, 32, 37, 47, 48, 52, 53, 55, 57, 72, 80, 81, 83, 84, 92, 96, 97, 99, 101, 104, 105, 108, 113, 120, 122–125, 128, 139]
Treatments - Medication - Expert-led treatments (conventional and CAM) - Self-care/self-management^a^ - Other^b^	28	[17, 19, 24, 25, 27, 31, 37, 48, 52, 53, 57, 58, 72, 80, 83, 84, 95, 96, 99, 100, 104, 105, 108, 113, 117, 122, 128, 131]
Screening and testing - for a specific condition - general health check	6	[17, 19, 27, 83, 96, 113]
For other types of health information^c^	4	[72, 83, 117, 122]
To acquire/develop resources for psycho-social support (n = 9)		
To gain reassurance, comfort and support including from others with lived or personal experience of the condition^d^	7	[23, 24, 37, 53, 94, 97, 117]
To gain a sense of control, ability to cope with the diagnosis, condition or treatment	4	[53, 69, 100, 117]
To navigate their own health journeys and the healthcare system (n = 31)		
To find information on or locate appropriate local healthcare providers	15	[17, 19, 20, 24, 27, 31, 48, 53, 68, 72, 80, 83, 125, 128, 131]
To prepare ahead of meeting HCPs / HC institutions^e^	5	[53, 68, 69, 95, 113]
To make health decisions, including whether to seek professional help	8	[19, 38, 53, 68, 72, 95, 117, 128]
To avoid going to a HC provider	1	[105]
To make own diagnosis, prevent or cure or manage disease /condition or maintain health	9	[19, 20, 45, 47, 68, 72, 104, 105, 145]
To verify/confirm/clarify or add to information received from another given source including: - To verify information from HCP (*n* = 6) - To obtain additional information^f^ (*n* = 3) - To clarify/understand info from HCP or medication label or prescriptions (n = 4) - General or unspecified (*n* = 1)	10	[20, 23, 37, 53, 55, 72, 93, 97, 103, 117]

^a^This includes, for example, home remedies, tips on what’s worked well for someone else with the condition

^b^This includes advice on caring for an elderly person, psychological care or unspecified treatments

^c^This includes information relating to health insurance, policies, and guidelines

^d^This may involve seeking support from patient groups, other families with children with a similar problem, or reading testimonials online

^e^For instance, in order to learn what questions to ask a healthcare professional or how to approach healthcare providers (e.g. importance of being persistent), to be one’s own health advocate

^f^For example because the individual did not have time to ask during their appointment with a healthcare professional or was afraid to ask

Fourteen included studies reported the ways which the HRI accessed was used by members of the public (Table 4). Reasons for use included: to improve participants’ own health behaviours and/or ability to manage their health (*n* = 4); to support health-related decision making (*n* = 5); to facilitate or enhance conversations or encounters with HCPs (*n* = 4); to increase people’s own understanding of a health-related topic (*n* = 3); to assess the information from another source (*n* = 2); and to share with or educate others in the context of providing psychosocial support (*n* = 1).

**Table 4:** Reported use/utilisation of accessed health research or information (HRI)

	Number of studies	Study number
Improve their own health behaviours or ability to manage their health^a^	4	[64, 125, 142, 144]
Make health decisions^b^	5	[57, 64, 66, 69, 104]
Facilitate / enhance conversations or encounters with healthcare professionals	4	[38, 53, 64, 141]
Increase their own understanding of a health-related matter^c^	3	[104, 125, 144]
Assess the information from another source	2	[108, 132]
Share / educate others in the context of providing psychosocial support	1	[131]

^a^Including, for example, developing better coping strategies or lower thresholds for seeking help

^b^This may include decision to change medication without discussing it with a healthcare professional

^c^Including for example, a dependent’s condition; own symptoms, treatment options, best use of insurance

### Factors influencing access to and use of health research and information

#### Barriers to accessing and using health research or information

Thirty studies reported barriers to accessing and using HRI. The main barriers related to: (i) the source characteristics (*n* = 24); (ii) the characteristics of the person accessing or using HRI (*n* = 12); the nature of the condition for which HRI was desired (*n* = 3). Other barriers such as a fear that seeking information could be distressing, inability to determine the quality of information appeared in seven studies (Table 5).

**Table 5:** Barriers to accessing and/or using health research or information

	No. of studies	Study number
Barriers relating to the characteristics of the source (channel, format) (*n* = 24)		
Language - Information not in preferred language (including national, local and sign languages) - Information not available in formats suitable for the visually impaired - Terminology / language used by or in channel is difficult to understand	17	[19, 20, 22, 23, 32, 38, 45, 88, 106–108, 116, 118–120, 127, 136]
Channel* availability - Expense of channel or cost of using channel - Preferred channel does not exist for specific condition or concern - Preferred channel (e.g. healthcare professional, pharmacist) is not easily available	11	[19, 20, 31, 32, 45, 72, 112, 116, 118, 121]
Quantity, quality and tone of information - Too much information is given - Information is too general, not explicit - Information is too impersonal - Information is inadequate, outdated or irrelevant	6	[19, 31, 45, 72, 94, 105]
Credibility—Channel is not trusted	1	[140]
Barriers related to the characteristics of the health research or information seeker (*n* = 12)		
Individual lacks personal resources that would enable effective health research or information access and use^a^	8	[19, 45, 69, 72, 106, 111–113]
Individual’s health or other physical characteristics^b^	2	[26, 107]
Age or other characteristic restricts access to sources of health research or information^c^	2	[94, 118]
Lack of awareness of sources of HRI on given condition/health topic	1	[19]
Barriers related to the nature of the condition for which health research or information is desired (*n* = 3)		
Condition is stigmatising or may lead to discrimination, concerns about disclosure^d^	3	[31, 32, 130, 130]
Other barriers (*n* = 7)		
Reluctance to search for information from fear it could be distressing	3	[69, 113, 131]
Inability to determine the quality of information of the source /poor info evaluation skills	3	[20, 32, 72]
Poor experiences with healthcare profession in the past	1	[140]

^*^Channel means the medium e.g. journal, website, radio programme, etc.

^a^This includes lack of technical or other skills, language, information retrieval, literacy, health literacy and time

^b^This may include, for example, visual impairment, deafness or limb amputation

^c^For example, restricted access to internet among youth by parents, or reliance on family members to access preferred channel

^d^For example, HIV/AIDS, depression, puberty, menstruation

#### Factors that facilitate accessing and using health research and information

Six studies discussed factors that facilitated members of the public access and use of HRI. Six studies reported factors related to the source of information that facilitated access to HRI. These included ease of access [120, 124, 142], anonymity [125, 142], cost [142], format and language in which HRI was presented [117, 120], and quantity and complexity of contents [128]. Factors facilitating access were: reports that did not use technical terms and acronyms but ‘sound[ed] scientific’ [117]; on-demand availability of the channel [120, 124, 142]; information that was up-to-date and provided both an outline of the topic and detail [128].

#### Factors influencing choice of source of health research and information

Three studies reported the factors that influenced people’s choice of source of HRI. Two studies found that the health condition searched for, and how it was perceived (i.e. trivial or stigmatising) influenced choice of source [103, 115]. One study reported that presenting health condition could influence choice [125]; one study noted that the healthcare provision available to study participants influenced choice of source [103]; and one study highlighted that patterns of access and use of HRI differed according to when in the patient journey this information was sought, and according to the purpose (for instance, the internet was not considered useful for making health decisions but it was useful for other health-related reasons) [115].

## Discussion

This scoping review was the first to be conducted with the aim to identify the extent and nature of the research literature on how members of the public access and use high quality health research and information.

The scoping review identified 130 studies that investigated how members of the public accessed HRI. Mass media was the most studied source of information, followed by printed information and the internet. The reasons for members of the public accessing and using HRI included to improve health behaviours, and/or ability to manage their health, to help with health-related decision making, facilitating or enhancing conversations or encounters with healthcare professionals, increasing people’s own understanding of a health-related topic; assessing the information from another source, and sharing with or educating others in the context of providing psychosocial support. The factors that constrained access and use of HRI, related to the source characteristics, the characteristics of the person accessing the HRI and the nature of the condition for which HRI was accessed. Six studies reported on the factors facilitating access and use of HRI, and three studies discussed factors that influenced the choice of one source rather than another.

### Health information vs health research

The review identified a substantial literature on broader concept of ‘health information’ but limited reporting of the general public’s utilisation of health research.

Crucially, only two included studies investigated access of health research by members of the public, and none of the included studies explored the use of health research by members of the public. One case study conducted in the USA found that a library of brief podcasts on health research (duration 22 min each) was feasible to co-produce with local community partners and generated user views /engagement over 18 months [15]. However, this preliminary study, conducted in a single state in the USA, did not specify the number of study participants and their demographics, limiting learnings from the study, as well as the generalisability and transferability of its findings. Another mixed-methods study investigated the relationship between information sources and public trust in health research in two European countries (Italy, Slovakia) [16]. In this study, traditional media (e.g. television, newspapers) and digital media (e.g. blogs, social networks) were the most widely cited information channels, followed by personal interaction and exchanges (e.g. family, friends, experts, people in authority), echoing the overall results of this scoping review. At ten roundtable discussions participants (*n* = 192) reported obtaining credible health research from a source considered authoritative and competent (e.g. health professionals). The experts provided the information needed to help the individual understand and evaluate complex issues via direct interaction. Taken together, these two studies suggest that the public will engage with health research in diverse ways and that delivery by a source perceived as competent or authoritative may be important to engagement with health research, whatever the medium.

All other included studies centred on the broad concept of ‘health information’. This potentially obscures the interest among the general public in accessing research evidence. For example, 16 included studies reported ‘scholarly/academic sources’ as a source of HRI, potentially indicating direct access to health research by members of the public (Table 2). This is supported by a recent mixed-methods study conducted by the UK’s National Institute of Health and Care Research, which found a strong interest among the general public in being able to access research findings [12]. However, neither the NIHR study nor the majority of studied mentioning scholarly/academic sources provide demographic data or disaggregated demographic data for the participants accessing and using these sources. Furthermore, the two included studies that highlight the use of scholarly sources of HRI and also provide relevant participant data [121, 122], suggest that such sources are more prevalent among more educationally privileged groups: in these two studies, up to 90–100% of study participants were college or university educated. It does not follow, however, that only more educated groups tend to access health research through scholarly or academic sources. Indeed, as studies such as Vandrevala et al. (forthcoming) have shown, information access and use is often a social act, with members of the public not only seeking information for themselves but others within their social network. The paucity of research on how members of the public access and use health research evidence, and the use of the umbrella term, ‘health information’, without explicit definition and distinguishing between the types of ‘health information’ sought, may underestimate the extent of access and use of research evidence, among the general public. The issue of paywalls excluding the general public from access to academic or scholarly sources such as journals was not raised in the retrieved literature.

Another issue highlighted by this review concerns the similarities and differences between how the general public and policymakers and practitioners use health research and HRI, respectively, though this will need further exploration. Like practitioners and policymakers, the general public’s uses included conceptual and instrumental uses of HRI [5]. In addition, the general public used HRI to obtain or provide psychosocial support, a use that was not noted in relation to research use by practitioners and policymakers.

### A vast diversity of ways of accessing health research or information

Included studies reported a wide range sources to access HRI, with at least 84 different sources identified, which were classified into three broad categories: ‘other people’, ‘professional settings’ (medical, community or educational places), and ‘independent searches’ (that covered all those tools that people use to do their own ‘research’ to access the information that they need). The review found that, even as interest in the internet and social media as means to access or deliver HRI has increased (e.g. [146, 147]), ‘traditional’ sources of information such as mass media or printed material are still relevant. For example, a 2016 survey conducted among Asian American groups in New York City (*n* = 1373), USA, found that the internet was among the least used sources of HRI, with print media being the most used source [46]. Similarly, a 2021 survey among cancer patients (*n* = 404) in Japan found the most widely used source of HRI to be newspapers, followed by healthcare professionals, and that the internet was used by a small proportion of the patients only [65]. These examples are not unique, and hint that *diversification of means of delivering HRI* to support self-care may be a more suitable approach for delivering HRI, though this conclusion is tentative and will need confirmation through a more systematic study and further research.

Communications technology has advanced rapidly in the past decade, notably through the increase in the number of internet platforms and the development of new functionalities so that, for instance, YouTube is no longer just a means to share video material but also features discussion boards. Instagram as a means to access HRI was mentioned in only one study [55], there was an absence of studies evaluating the role of Tiktok, a popular channel [148], and social media influencers as ways to deliver HRI (e.g. [149]), suggesting that this literature is now dated. Equally, podcasts were infrequently mentioned in the included studies, in spite of their growing appeal as a way to disseminate medical knowledge [150].

In addition, many studies lacked detail. For instance, studies reported ‘online chatrooms’ as a source of information without specifying the platform for the chatroom, whether social media or a specialist health organisation. Some sources of information such as social media were insufficiently distinguished in studies, for example Twitter and Instagram, which tend to favour one or the other format and may therefore appeal to different audiences. Generally, very few included studies considered or reported on the format of the HRI accessed.

### Barriers and facilitators to independent searches vs other sources of health research or information

Included studies did not generally explore barriers and facilitators to the use of HRI, or, if they did, they did not report barriers to use separately from barriers to access. This section focuses therefore on barriers to and facilitators of access.

The studies included in this review described a wide range of factors that shaped how the public accessed HRI. These were classified into 16 different factors under four overarching categories that related to personal characteristics, source characteristics and nature of the health condition of interest or presenting and ‘other’ factors.

Relating these to the sources of HRI identified in this review (‘other people’, ‘professional settings’ and ‘independent searches’), included studies provided a detailed understanding of barriers to access and, in particular, barriers to access through *independent searches,* where major considerations related to how information is presented, namely: the format, the language used, the quantity of information and the level of detail provided. There was no consensus among studies, however, with some identifying as facilitators shorter pieces in simple, non-technical language while others indicated that accessible but ‘scientific-sounding’ (including some level of technical language) and more detailed information facilitated access to HRI.

Only one barrier was identified that related to ‘other people’ as sources of HRI, and that concerned the availability of the source. None of the studies specifically identified barriers relating to ‘professional settings’, though conceivably, features of the setting, including its physical features, may act as a barrier to accessing HRI. One example was provided by a study of people with autism which reported struggling with the physical environment of specialist clinics [151].

Studies provided a good understanding of the characteristics of the individual seeking information that may act as a barrier to accessing HRI, mainly their possession of specific technical skills (technological, linguistic, information retrieval) and time. However, again, these pertained mostly to independent searches rather than accessing HRI through other sources. No mention was made of the cultural knowledge and skills needed to navigate the professional settings or relationships through which HRI may be accessed, although it is known that lack of familiarity with healthcare systems and its norms can be a barrier to accessing these settings (e.g. [152]), and therefore, potentially, HRI.

Another factor shaping how people accessed HRI that was seldom investigated in included studies was the role of *past experience with healthcare services, either an individual’s own lived experience of these services or that of other members of their community* or social network. This was reported in one included study only [140], and in relation to a specific community (Lesbian, Gay and Bisexual adolescents). This absence is surprising, given the evidence that negative experiences with healthcare provision will impact health behaviours (e.g. [153]) and that negative experiences in the community will impact information seeking generally (e.g. [154]).

In a systematic review including 344 studies, Mirzaei et al. [6] identified a total of 1595 significant ‘predictors of health information seeking behaviours’, (defined as the variables affecting the actions of seeking out information) and classified these into 67 different categories. Although health information seeking behaviour and accessing and using HRI are not identical conceptually, there were parallels between the current scoping review findings and Mirzaei et al.’s [6] comprehensive typology. In addition, this scoping review built on Mirzaei et al.’s [6] findings: while Mirzaei et al. [6] had identified the role of previous exposure to a healthcare source of information as a predictor of health information seeking behaviour, this review identified that past lived experience with healthcare services generally (whether or not it was a source of information) in shaping how members of the public accessed HRI. Given the differences between this scoping review and Mirzaei et al. [6]’s systematic review, it is not possible to draw firm conclusions regarding influences on accessing different types of health information (Mirzaei et al.’s [6] definition is broader) or differences across groups (Mirzaei et al. [6] include the general public as well as healthcare practitioners and healthcare students). This will need further detailed exploration.

### Limitations

Due to funding and time constraints this review only included peer-reviewed studies published in English language between 01/01/2010 and 18/01/2022. No grey literature searches or manual searching of the reference lists of included studies were conducted. However, we searched the reference lists of relevant systematic reviews and meta-analysis, and consulted experts in the field to ensure that very few, if any, relevant studies produced during this period had been overlooked. Studies published since January 2022, unpublished studies or studies in other languages, though, will not have been captured.

Limiting the review to English language studies may have influenced in the geographical bias of included literature, with a majority of studies conducted among North American populations. However, evidence indicates that the conclusions of most systematic reviews are not altered through the omission of non-English language studies, and the exclusion of non-English language publications aligns with recommendations from the Cochrane collaboration [155].

The conclusions from this review were hampered by poor reporting in some included studies particularly the lack of clear definitions for the term ‘health information’. As a result this review may have included studies with a broader definition of ‘health information’, though this is likely to apply in a very small number of cases only.

### Implications

This scoping review found a lack of research on research use by members of the public. This absence may not reflect the extent to which the public uses research, given the subset of studies identifying scholarly sources as a means to access HRI by the general public in this review, and the fact that people will often access HRI on others’ behalf in their communities or social networks. This justifies more primary research in this area or a detailed review focusing on this subset, including contacting authors for more information on their study. Research on research access and use by the general public could also usefully explore the differences in access and use between the general public and practitioners and policymakers, for instance, through a systematic review including grey literature and increased number of databases consulted.

The review also identified the need for an update on the barriers in accessing HRI, following the observation that barriers (e.g. cost of internet access) have considerably decreased for some groups in the last decade. More specifically, it highlighted a need to enrich current knowledge of the facilitators of both HRI *access and use* and barriers to *use* of HRI, in relation to the following:

- The factors shaping access to HRI *through ‘other people’ and ‘professional settings’*, with specific attention to features of the setting and the presence or absence of cultural skills to navigate the professional settings where HRI is accessed;
- A better understanding of the role of *lived experience of individuals or communities* with healthcare providers in shaping access to HRI;
- A better understanding of person and setting characteristics that *facilitate* access to HRI
- A better understanding generally of the factors shaping how the public *uses* HRI.

Finally, the literature was found to be dated in relation to the sources of HRI explored, underscoring the need for primary research to update our knowledge of the communications tools currently in use among different populations, and the formats that are now being adopted by social media networking platforms (e.g. Instagram in-feed, stories, and reels; YouTube Community Tab).

## Conclusions

This scoping exercise, the first to adopt a narrow definition of health information in an attempt to understand how the public accesses and uses ‘high quality health and care information’, identified major patterns of access and use and also identified gaps in the existing research literature. Major patterns included: the use of a wide diversity of sources to access HRI, with traditional sources still relevant alongside newer sources; access and use for HRI a wide range of reasons, from the conceptual to the psychosocial, both for self and for others. Barriers to use related to how HRI is presented (e.g. language, quantity of information and level of detail) and its availability; the skill, knowledge and time of the person accessing the information, their physical condition and autonomy; and the perception of a health topic or the personal and social implications of searching a given topic. Gaps in the evidence included: a limited number of studies focussing on how members of the public accesses health research and how the public uses health research; the absence of newer (online) sources of HR/I, and the lack of exploration of the features and functionalities of online sources. The review also identified that there is a need for more detailed studies on the factors that shape how the public *access* HRI through other people and by visiting professional settings. Primary research investigating the factors that shape how the public *uses* health research and information is also needed, notably, by paying more attention to lived experience of healthcare provision and the cultural knowledge that is required by the public when attempting to access certain sources of health information.

Finally the review found that, given the challenges around reporting and the lack of precise definition of the term ‘information’, identifying how the public accesses and uses high quality information is not straightforward at present. More precise definitions of the term ‘information’, and studies based on these will be needed to find ways for policy-makers to better support self-care and improve health outcomes among the general public.

### Supplementary Information

**Additional file 1:**

**Additional file 2: Supplementary table 1.** Search strategy.

**Additional file 3: Supplementary table 2.** List of included studies, showing relevance to scoping review objective and evidence.

## Data Availability

The datasets used and/or analysed during the current study are available from the corresponding author on reasonable request.

## Abbreviations

HCP: Healthcare professional or provider
HISB: Health information seeking behaviour
HRI: Health research or information
