# Supplementary material for: Exploring how members of the public access and use health research and information: a scoping review
Source: BMC Public Health. 2023 Nov 7;23:2179. doi: 10.1186/s12889-023-16918-8 (PMC10629152; doi:10.1186/s12889-023-16918-8)
Supplement: Supplementary file 2 — Additional file 2: Supplementary table 1. Search strategy. [file 12889_2023_16918_MOESM2_ESM.docx]

# Supplementary table 1 – Search strategy

**Databases**: CINAHL Plus, Medline, PsycInfo, Social Sciences Full text Search Strategy (combined on EBSCO platform)

**Abstract and title only**

**Limits**: English only; 01-01-2010 to 18-01-2022 only; academic journals, peer-reviewed articles

**Searches**:

##### Search 1 - ‘health information’

a) “access to health information” OR “use of health information” OR “utilization of health information” OR “accessing health information” OR “using health information” OR “utilizing health information” OR “health information utilization” OR “uptake of health information” OR “health information uptake” OR “adoption of health information” OR “adopting health information” **(1859 hits)**

b) public OR general public OR people OR community (index terms) OR lay public (text words) OR patient OR carer OR lay person (**6,096,162 hits**)

**a) AND b) 1272 hits**

##### Search 2 - ‘health research’

a) “access to health research” OR “use of health research” OR “utilization of health research” OR “accessing health research” OR “using health research” OR “utilizing health research” OR “health research utilization” OR “uptake of health research” OR “health research uptake” OR “adoption of health research” OR “adopting health research” (**92 hits**)

b) public OR “general public” OR people OR community OR “lay public” OR patient OR carer OR “lay person”

a) AND b) **30** **hits**

##### Search 3 - ‘Research evidence’

a) “access to research evidence” OR “use of research evidence” OR “utilization of research evidence” OR “accessing research evidence” OR “using research evidence” OR “utilizing research evidence” OR “research utilization” OR “uptake of research evidence” OR “research evidence uptake” OR “research uptake” OR “adoption of research evidence” OR “adopting research evidence” (all text words, except ‘research utilization’, index term) ( **947 hits**)

b) health OR healthcare OR “health care” (exploded MeSH term) (**2,238,037 hits**)

c) public OR “general public” OR people OR community OR “lay public” OR patient OR carer OR “lay person”

**a) AND b) AND c): 302 hits**

##### Search 4 - ‘Scientific evidence’

a) “access to scientific evidence” OR “use of scientific evidence” OR “utilization of scientific evidence” OR “accessing scientific evidence” OR “using scientific evidence” OR “utilizing scientific evidence” OR “scientific evidence utilization” OR “uptake of scientific evidence” OR “scientific evidence uptake” OR “scientific evidence uptake” OR “adoption of scientific evidence” OR “adopting scientific evidence” (**147 hits**)

b) health OR healthcare OR “health care”

c) public OR “general public” OR people OR community OR “lay public” OR patient OR carer OR “lay person”

**a) AND b) AND c): 52 hits**

##### Search 5 - ‘research findings’

a) “access to research findings” OR “accessing research findings” OR “use of research findings” OR “utilization of research findings” OR “using research findings” OR “access to scientific findings” OR “accessing scientific findings” OR “use of scientific findings” OR “utilization of scientific findings” OR “using scientific findings” (**470 hits**)

b) health OR healthcare OR “health care”

c) public OR “general public” OR people OR community OR “lay public” OR patient OR carer OR “lay person”

**a) AND b) AND c): 46 hits**

##### Search 6 - ‘research outputs’

a) “access to research outputs” OR “accessing research outputs” OR “use of research outputs” OR “utilization of research outputs” OR “using research outputs” OR “access to scientific outputs” OR “accessing scientific outputs” OR “use of scientific outputs” OR “utilization of scientific outputs” OR “using scientific outputs” (**5 hits**)

b) health OR healthcare OR “health care”

c) public OR “general public” OR people OR community OR “lay public” OR patient OR carer OR “lay person”

**a) AND b) AND c): 1 hit**

##### Search 7 - research or scientific publications/articles

a) “access to scientific articles” OR “access to research articles” OR “access to research publications” OR “access to scientific publications” OR “accessing scientific articles” OR “accessing research articles” OR “accessing research publications” OR “accessing scientific publications” (**24 hits**)

b) health OR healthcare OR “health care”

c) public OR “general public” OR people OR community OR “lay public” OR patient OR carer OR “lay person”

**a) AND b) AND c): 4 hits**

##### Search 8 – Engagement

a) “engagement with research” OR “research engagement” OR “engagement with science” OR “science engagement” (**1457 hits**)

b) health OR healthcare OR “health care”

c) public OR “general public” OR people OR community OR “lay public” OR patient OR carer OR “lay person”

**a) AND b) AND c): 535 hits**

##### Search 9 – other search terms

a) “access to scientific knowledge” OR “access to research” OR “research accessibility” (**484 hits**)

b) health OR healthcare OR “health care”

c) public OR “general public” OR people OR community OR “lay public” OR patient OR carer OR “lay person”

**a) AND b) AND c): 122 hits**

**Total EBSCO (CINAHL, PsycInfo, MedLine, Social Science Full Text): 2364**

**________________________________________________________________________________**

**Database**: SCOPUS

**Abstract and title only**

**Limits**: English only; 01-01-2010 to 18-01-2022 only; articles only

##### Search 1 - ‘health information’

a) TITLE-ABS({access to health information} OR {use of health information} OR {utilization of health information} OR {accessing health information} OR {using health information} OR {utilizing health information} OR {health information utilization} OR {uptake of health information} OR {health information uptake} OR {adoption of health information} OR {adopting health information}) (**908 hits**)

b) TITLE-ABS(public OR {general public} OR people OR community OR {lay public} OR patient OR carer OR {lay person}) (**4,691,978**  **hits**)

a) AND b) **647 hits**

##### Search 2 - ‘health research’

1. TITLE-ABS({access to health research} OR {use of health research} OR {utilization of health research} OR {accessing health research} OR {using health research} OR {utilizing health research} OR {health research utilization} OR {uptake of health research} OR {health research uptake} OR {adoption of health research} OR {adopting health research}) (**17** **hits**)

b) TITLE-ABS(public OR {general public} OR people OR community OR {lay public} OR patient OR carer OR {lay person})

a) AND b) **10 hits**

##### Search 3 - ‘Research evidence’

1. TITLE-ABS({access to research evidence} OR {use of research evidence} OR {utilization of research evidence} OR {accessing research evidence} OR {using research evidence} OR {utilizing research evidence} OR {research utilization} OR {uptake of research evidence} OR {research evidence uptake} OR {research uptake} OR {adoption of research evidence} OR {adopting research evidence})  **537 hits**

b) TITLE-ABS(health OR healthcare OR {health care}) **1,512,118 hits**

c) TITLE-ABS(public OR {general public} OR people OR community OR {lay public} OR patient OR carer OR {lay person}) **4,691,978 hits**

a) AND b) AND c):  **148 hits**

##### Search 4 - ‘Scientific evidence’

a) TITLE-ABS({access to scientific evidence} OR {use of scientific evidence} OR {utilization of scientific evidence} OR {accessing scientific evidence} OR {using scientific evidence} OR {utilizing scientific evidence} OR {scientific evidence utilization} OR {uptake of scientific evidence} OR {scientific evidence uptake} OR {scientific evidence uptake} OR {adoption of scientific evidence} OR {adopting scientific evidence}) (**95 hits**)

b) TITLE-ABS(health OR healthcare OR {health care})

c) TITLE-ABS(public OR {general public} OR people OR community OR {lay public} OR patient OR carer OR {lay person})

a) AND b) AND c): **31 hits**

##### Search 5 - ‘research findings’

a) TITLE-ABS({access to research findings} OR {accessing research findings} OR {use of research findings} OR {utilization of research findings} OR {using research findings} OR {access to scientific findings} OR {accessing scientific findings} OR {use of scientific findings} OR {utilization of scientific findings} OR {using scientific findings}) ( **132 hits**)

b) TITLE-ABS(health OR healthcare OR {health care}) (**1,512,118 hits, as above)**

c) TITLE-ABS(public OR {general public} OR people OR community OR {lay public} OR patient OR carer OR {lay person})

a) AND b) AND c): **19 hits**

##### Search 6 - ‘research/scientific outputs’

1. TITLE-ABS({access to research outputs} OR {accessing research outputs} OR {use of research outputs} OR {utilization of research outputs} OR {using research outputs} OR {access to scientific outputs} OR {accessing scientific outputs} OR {use of scientific outputs} OR {utilization of scientific outputs} OR {using scientific outputs}) ( **11 hits**)

b) TITLE-ABS(health OR healthcare OR {health care})

c) TITLE-ABS(public OR {general public} OR people OR community OR {lay public} OR patient OR carer OR {lay person})

a) AND b) AND c):  **0 hits**

##### Search 7 - research or scientific publications/articles

a) TITLE-ABS({access to scientific articles} OR {access to research articles} OR {access to research publications} OR {access to scientific publications} OR {accessing scientific articles} OR {accessing research articles} OR {accessing research publications} OR {accessing scientific publications}) ( **26 hits**)

b) TITLE-ABS(health OR healthcare OR {health care})

c) TITLE-ABS(public OR {general public} OR people OR community OR {lay public} OR patient OR carer OR {lay person})

a) AND b) AND c):  **6 hits**

##### Search 8 – Engagement

a) TITLE-ABS({engagement with research} OR {research engagement} OR {engagement with science} OR {science engagement}) ( **793 hits**)

b) TITLE-ABS(health OR healthcare OR {health care})

c) TITLE-ABS(public OR {general public} OR people OR community OR {lay public} OR patient OR carer OR {lay person})

a) AND b) AND c): **110 hits**

##### Search 9 – other search terms

a) TITLE-ABS({access to scientific knowledge} OR {access to research} OR {research accessibility}) ( **463 hits**)

b) TITLE-ABS(health OR healthcare OR {health care})

c) TITLE-ABS(public OR {general public} OR people OR community OR {lay public} OR patient OR carer OR {lay person})

a) AND b) AND c): **71 hits**

**Total SCOPUS: 1042**

**________________________________________________________________________________**

Web Of Science search (Editions = Science Citation Index Expanded (SCI-EXPANDED), Social Sciences Citation Index (SSCI), Emerging Sources Citation Index (ESCI))

**Topic only (Title, abstract and keywords)**

**Limits**: English only; 01-01-2010 to 18-01-2022 only; academic journals, peer-reviewed articles

##### Search 1 - ‘health information’

a) TS=(“access to health information” OR “use of health information” OR “utilization of health information” OR “accessing health information” OR “using health information” OR “utilizing health information” OR “health information utilization” OR “uptake of health information” OR “health information uptake” OR “adoption of health information” OR “adopting health information”) (**725 hits**)

b) TS=(public OR “general public” OR people OR community OR “lay public” OR patient OR carer OR “lay person”) (**4,214,173 hits**)

a) AND b) **553 hits**

##### Search 2 - ‘health research’

a) TS=(“access to health research” OR “use of health research” OR “utilization of health research” OR “accessing health research” OR “using health research” OR “utilizing health research” OR “health research utilization” OR “uptake of health research” OR “health research uptake” OR “adoption of health research” OR “adopting health research”) ( **hits**)

b) TS=(public OR "general public" OR people OR community OR "lay public" OR patient OR carer OR "lay person")

a) AND b) **8 hits**

##### Search 3 - ‘Research evidence’

a) TS=(“access to research evidence” OR “use of research evidence” OR “utilization of research evidence” OR “accessing research evidence” OR “using research evidence” OR “utilizing research evidence” OR “research utilization” OR “uptake of research evidence” OR “research evidence uptake” OR “research uptake” OR “adoption of research evidence” OR “adopting research evidence”) (**604 hits**)

b) TS=(health OR healthcare OR “health care”) (**1,517,557 hits**)

c) TS=(public OR "general public" OR people OR community OR "lay public" OR patient OR carer OR "lay person")

a) AND b) AND c): **185 hits**

##### Search 4 - ‘Scientific evidence’

a) TS=(“access to scientific evidence” OR “use of scientific evidence” OR “utilization of scientific evidence” OR “accessing scientific evidence” OR “using scientific evidence” OR “utilizing scientific evidence” OR “scientific evidence utilization” OR “uptake of scientific evidence” OR “scientific evidence uptake” OR “scientific evidence uptake” OR “adoption of scientific evidence” OR “adopting scientific evidence”) (**79 hits**)

b) TS=(health OR healthcare OR “health care”)

c) TS=(public OR "general public" OR people OR community OR "lay public" OR patient OR carer OR "lay person")

a) AND b) AND c): **29 hits**

##### Search 5 - ‘research findings’

a) TS=(“access to research findings” OR “accessing research findings” OR “use of research findings” OR “utilization of research findings” OR “using research findings” OR “access to scientific findings” OR “accessing scientific findings” OR “use of scientific findings” OR “utilization of scientific findings” OR “using scientific findings”) (**98 hits**)

b) TS=(health OR healthcare OR health care)

c) TS=(public OR "general public" OR people OR community OR "lay public" OR patient OR carer OR "lay person")

a) AND b) AND c): **21 hits**

##### Search 6 - ‘research outputs’

a) TS=(“access to research outputs” OR “accessing research outputs” OR “use of research outputs” OR “utilization of research outputs” OR “using research outputs” OR “access to scientific outputs” OR “accessing scientific outputs” OR “use of scientific outputs” OR “utilization of scientific outputs” OR “using scientific outputs”) (**6 hits**)

b) TS=(health OR healthcare OR health care)

c) TS=(public OR "general public" OR people OR community OR "lay public" OR patient OR carer OR "lay person")

a) AND b) AND c): **0 hits**

##### Search 7 - research or scientific publications/articles

a) TS=(“access to scientific articles” OR “access to research articles” OR “access to research publications” OR “access to scientific publications” OR “accessing scientific articles” OR “accessing research articles” OR “accessing research publications” OR “accessing scientific publications”) (**22 hits**)

b) TS=(health OR healthcare OR health care)

c) TS=(public OR "general public" OR people OR community OR "lay public" OR patient OR carer OR "lay person")

a) AND b) AND c): **3 hits**

##### Search 8 – Engagement

a) TS=(“engagement with research” OR “research engagement” OR “engagement with science” OR “science engagement”) (**830 hits**)

b) TS=(health OR healthcare OR health care)

c) TS=(public OR "general public" OR people OR community OR "lay public" OR patient OR carer OR "lay person")

a) AND b) AND c): **139 hits**

##### Search 9 – other search terms

a) TS=(“access to scientific knowledge” OR “access to research” OR “research accessibility”) (**353 hits**)

b) TS=(health OR healthcare OR health care)

c) TS=(public OR "general public" OR people OR community OR "lay public" OR patient OR carer OR "lay person")

a) AND b) AND c): **66 hits**

**Total Web of Science: 1,004 hits**

**Total database search prior to de-duplication: 4410**
