# Supplementary material for: Exploring how members of the public access and use health research and information: a scoping review
Source: BMC Public Health. 2023 Nov 7;23:2179. doi: 10.1186/s12889-023-16918-8 (PMC10629152; doi:10.1186/s12889-023-16918-8)
Supplement: Supplementary file 3 — Additional file 3: Supplementary table 2. List of included studies, showing relevance to scoping review objective and evidence. [file 12889_2023_16918_MOESM3_ESM.docx]

## Supplementary Table 2 – List of included studies, showing relevance to scoping review objective and evidence

| **Author & date** | **Study type** | **Methodology** | **Participant numbers** | **Participant character-ristics** | **Participant location** | **Reasons for access** | **Source of HR/I** | **Format of HR/I** | **Use of HR/I** | **Barriers**  **& facili-tators** |
| --- | --- | --- | --- | --- | --- | --- | --- | --- | --- | --- |
| Abara et al 2010 | ✔️ | ✔️ | ✔️ | ✔️ | ✔️ | ✔️ | ✔️ |  |  |  |
| Afeef et al 2021 | ✔️ | ✔️ | ✔️ | ✔️ | ✔️ | ✔️ | ✔️ |  |  |  |
| Ahmad et al 2021 | ✔️ | ✔️ | ✔️ | ✔️ | ✔️ | ✔️ | ✔️ | ✔️ |  | ✔️ |
| Ahola et al 2017 | ✔️ | ✔️ | ✔️ | ✔️ | ✔️ | ✔️ | ✔️ |  |  | ✔️ |
| Akidi 2019 | ✔️ | ✔️ | ✔️ |  | ✔️ | ✔️ | ✔️ |  |  | ✔️ |
| AlGhamdi 2012 | ✔️ | ✔️ | ✔️ | ✔️ | ✔️ | ✔️ | ✔️ |  |  |  |
| Alhuwail et al 2018 | ✔️ | ✔️ |  |  | ✔️ |  | ✔️ |  |  |  |
| Almusawi et al 2021 | ✔️ | ✔️ | ✔️ | ✔️ | ✔️ |  | ✔️ |  |  | ✔️ |
| Alsaadi 2012 | ✔️ | ✔️ | ✔️ | ✔️ | ✔️ | ✔️ | ✔️ |  |  | ✔️ |
| Alvarez-Galvez et al 2020 | ✔️ | ✔️ | ✔️ | ✔️ | ✔️ | ✔️ | ✔️ | ✔️ |  |  |
| Anyaoku & Nwosu 2017 | ✔️ | ✔️ | ✔️ | ✔️ | ✔️ |  | ✔️ |  |  |  |
| Ashing-Giwa et al 2012 | ✔️ | ✔️ | ✔️ | ✔️ | ✔️ | ✔️ | ✔️ |  |  |  |
| Athanasopoulou et al 2017 | ✔️ | ✔️ | ✔️ | ✔️ | ✔️ |  | ✔️ |  |  | ✔️ |
| Balls-Berry et al 2018 | ✔️ | ✔️ |  |  | ✔️ |  | ✔️ | ✔️ |  |  |
| Benard & Chipungahelo 2017 | ✔️ | ✔️ | ✔️ | ✔️ | ✔️ | ✔️ | ✔️ |  |  |  |
| Bianco et al 2013 | ✔️ | ✔️ | ✔️ | ✔️ | ✔️ | ✔️ | ✔️ |  |  |  |
| Blaga et al 2019 | ✔️ | ✔️ | ✔️ | ✔️ | ✔️ |  | ✔️ |  |  | ✔️ |
| Brondi et al 2021 | ✔️ | ✔️ | ✔️ | ✔️ | ✔️ |  | ✔️ |  |  |  |
| Bulled 2011 | ✔️ | ✔️ | ✔️ | ✔️ | ✔️ | ✔️ | ✔️ |  |  | ✔️ |
| Chae & Quick 2015 | ✔️ | ✔️ | ✔️ | ✔️ | ✔️ |  | ✔️ |  |  |  |
| Chavarria et al 2017 | ✔️ | ✔️ | ✔️ | ✔️ | ✔️ | ✔️ | ✔️ |  |  |  |
| Chisolm et al 2011 | ✔️ | ✔️ | ✔️ | ✔️ | ✔️ |  | ✔️ |  |  |  |
| Cho et al 2011 | ✔️ | ✔️ | ✔️ | ✔️ | ✔️ | ✔️ | ✔️ | ✔️ |  |  |
| Chung et al 2020 | ✔️ | ✔️ | ✔️ | ✔️ | ✔️ |  | ✔️ |  |  |  |
| Clark et al 2014 | ✔️ | ✔️ | ✔️ |  | ✔️ |  | ✔️ |  |  |  |
| Coffey et al 2017 | ✔️ | ✔️ | ✔️ | ✔️ | ✔️ | ✔️ | ✔️ |  |  |  |
| Coffey et al 2016 | ✔️ | ✔️ | ✔️ | ✔️ | ✔️ | ✔️ | ✔️ |  |  |  |
| Colineau & Paris 2010 | ✔️ | ✔️ | ✔️ | ✔️ | ✔️ | ✔️ | ✔️ | ✔️ |  |  |
| Criss et al 2015 | ✔️ | ✔️ | ✔️ | ✔️ | ✔️ |  | ✔️ |  |  |  |
| Cutilli 2010 | ✔️ | ✔️ |  |  |  |  | ✔️ |  |  |  |
| Das et al 2015 | ✔️ | ✔️ | ✔️ |  | ✔️ |  | ✔️ |  |  |  |
| Donelle & Hall 2016 | ✔️ | ✔️ | ✔️ | ✔️ | ✔️ |  | ✔️ |  |  |  |
| Edewor 2010 | ✔️ | ✔️ | ✔️ | ✔️ | ✔️ | ✔️ | ✔️ | ✔️ |  | ✔️ |
| Esmaeilzadeh et al 2018 | ✔️ | ✔️ | ✔️ | ✔️ | ✔️ | ✔️ | ✔️ |  |  | ✔️ |
| Eysenbach et al 2014 | ✔️ | ✔️ | ✔️ | ✔️ | ✔️ |  | ✔️ |  |  |  |
| Faith et al 2016 | ✔️ | ✔️ | ✔️ | ✔️ | ✔️ | ✔️ | ✔️ | ✔️ |  |  |
| Feinstein 2014 | ✔️ | ✔️ | ✔️ | ✔️ | ✔️ | ✔️ | ✔️ |  | ✔️ |  |
| Filippi et al 2013 | ✔️ | ✔️ | ✔️ | ✔️ | ✔️ | ✔️ | ✔️ |  |  |  |
| Finney et al 2016 | ✔️ | ✔️ | ✔️ | ✔️ | ✔️ |  | ✔️ |  |  |  |
| Frey et al 2020 | ✔️ | ✔️ | ✔️ | ✔️ | ✔️ |  | ✔️ |  |  |  |
| Garg et al 2015 | ✔️ | ✔️ |  | ✔️ |  |  | ✔️ |  |  |  |
| Garrido et al 2019 | ✔️ | ✔️ | ✔️ | ✔️ | ✔️ | ✔️ | ✔️ |  |  |  |
| Gavgani et al 2013 | ✔️ | ✔️ | ✔️ | ✔️ | ✔️ | ✔️ | ✔️ |  |  |  |
| Geana et al 2012 | ✔️ | ✔️ | ✔️ | ✔️ | ✔️ | ✔️ | ✔️ | ✔️ | ✔️ | ✔️ |
| Gonzalez et al 2019 | ✔️ | ✔️ | ✔️ | ✔️ | ✔️ | ✔️ | ✔️ |  |  |  |
| Greenstock et al 2013 | ✔️ | ✔️ | ✔️ | ✔️ | ✔️ |  | ✔️ |  |  |  |
| Greenstock et al 2012 | ✔️ | ✔️ | ✔️ | ✔️ | ✔️ |  | ✔️ |  |  |  |
| Hillyer et al 2017 | ✔️ | ✔️ | ✔️ | ✔️ | ✔️ |  | ✔️ |  |  |  |
| Hogan et al 2016 | ✔️ | ✔️ | ✔️ | ✔️ | ✔️ |  | ✔️ |  |  |  |
| Honey et al 2016 | ✔️ | ✔️ |  | ✔️ | ✔️ | ✔️ | ✔️ |  |  |  |
| Iorver 2020 | ✔️ | ✔️ | ✔️ | ✔️ | ✔️ | ✔️ | ✔️ |  |  | ✔️ |
| Islam et al 2016 | ✔️ | ✔️ | ✔️ | ✔️ | ✔️ |  | ✔️ |  |  |  |
| Jalilian et al 2021 | ✔️ | ✔️ | ✔️ | ✔️ | ✔️ | ✔️ | ✔️ |  |  |  |
| Jensen et al 2021 | ✔️ | ✔️ | ✔️ | ✔️ | ✔️ |  | ✔️ |  |  | ✔️ |
| Jo et al 2010 | ✔️ | ✔️ | ✔️ | ✔️ | ✔️ | ✔️ | ✔️ |  |  |  |
| Jones et al 2020 | ✔️ | ✔️ | ✔️ | ✔️ | ✔️ |  | ✔️ |  |  | ✔️ |
| Judd-Glossy et al 2022 | ✔️ | ✔️ | ✔️ | ✔️ | ✔️ | ✔️ | ✔️ | ✔️ | ✔️ | ✔️ |
| Kelley et al 2015 | ✔️ | ✔️ | ✔️ | ✔️ | ✔️ |  | ✔️ |  |  |  |
| Kim 2017 | ✔️ | ✔️ | ✔️ | ✔️ | ✔️ |  | ✔️ |  |  | ✔️ |
| Kim et al 2021 | ✔️ | ✔️ | ✔️ | ✔️ | ✔️ | ✔️ | ✔️ |  |  |  |
| Kim & Kwon 2010 | ✔️ | ✔️ | ✔️ | ✔️ | ✔️ |  | ✔️ |  |  |  |
| King-Shier et al 2018 | ✔️ | ✔️ | ✔️ | ✔️ | ✔️ | ✔️ | ✔️ |  | ✔️ | ✔️ |
| Koohkan et al 2019 | ✔️ | ✔️ | ✔️ | ✔️ | ✔️ |  | ✔️ |  |  |  |
| Kugbey et al 2019 | ✔️ | ✔️ | ✔️ | ✔️ | ✔️ | ✔️ | ✔️ |  |  |  |
| Lam et al 2015 | ✔️ | ✔️ | ✔️ | ✔️ | ✔️ |  | ✔️ |  | ✔️ |  |
| Laurent et al 2012 | ✔️ | ✔️ | ✔️ | ✔️ | ✔️ |  | ✔️ |  | ✔️ |  |
| Lee et al 2010 | ✔️ | ✔️ | ✔️ | ✔️ | ✔️ |  | ✔️ |  |  |  |
| Lim et al 2022 | ✔️ | ✔️ | ✔️ | ✔️ | ✔️ | ✔️ | ✔️ |  |  |  |
| Lustria et al 2010 | ✔️ | ✔️ | ✔️ | ✔️ | ✔️ |  | ✔️ |  |  |  |
| Maddock et al 2011 | ✔️ | ✔️ | ✔️ | ✔️ | ✔️ | ✔️ | ✔️ | ✔️ | ✔️ |  |
| Maitra & Rowley 2021 | ✔️ | ✔️ | ✔️ | ✔️ | ✔️ |  | ✔️ |  |  |  |
| Maraziene et al 2012 | ✔️ | ✔️ | ✔️ | ✔️ | ✔️ | ✔️ | ✔️ |  |  |  |
| Marco-Ruiz et al 2020 | ✔️ | ✔️ | ✔️ | ✔️ | ✔️ |  | ✔️ |  |  |  |
| Martensson et al 2020 | ✔️ | ✔️ | ✔️ | ✔️ | ✔️ |  | ✔️ | ✔️ |  | ✔️ |
| Masson et al 2019 | ✔️ | ✔️ | ✔️ | ✔️ | ✔️ |  | ✔️ |  |  |  |
| Messias & Estrada 2017 | ✔️ | ✔️ | ✔️ | ✔️ | ✔️ |  | ✔️ |  |  |  |
| Mi et al 2014 | ✔️ | ✔️ | ✔️ | ✔️ | ✔️ |  | ✔️ |  |  |  |
| Moorhead et al 2013 | ✔️ | ✔️ | ✔️ | ✔️ | ✔️ | ✔️ | ✔️ |  |  |  |
| Nangsangna & da-Costa 2019 | ✔️ | ✔️ | ✔️ | ✔️ | ✔️ |  | ✔️ |  |  |  |
| Neter & Brainin 2012 | ✔️ | ✔️ | ✔️ | ✔️ | ✔️ |  | ✔️ | ✔️ | ✔️ |  |
| Obaremi & Olatokun 2021 | ✔️ | ✔️ | ✔️ | ✔️ | ✔️ |  | ✔️ | ✔️ |  | ✔️ |
| Ohlow et al 2013 | ✔️ | ✔️ | ✔️ | ✔️ | ✔️ |  | ✔️ |  | ✔️ | ✔️ |
| Osei et al 2017 | ✔️ | ✔️ | ✔️ | ✔️ | ✔️ | ✔️ | ✔️ |  | ✔️ |  |
| Ozaki et al 2021 | ✔️ | ✔️ | ✔️ | ✔️ | ✔️ |  | ✔️ |  |  |  |
| Ozkan et al 2016 | ✔️ | ✔️ | ✔️ | ✔️ | ✔️ |  | ✔️ |  | ✔️ |  |
| Paige et al 2021 | ✔️ | ✔️ | ✔️ | ✔️ | ✔️ |  | ✔️ |  |  |  |
| Papen 2012 | ✔️ | ✔️ | ✔️ | ✔️ | ✔️ | ✔️ | ✔️ |  |  | ✔️ |
| Parija et al 2020 | ✔️ | ✔️ | ✔️ | ✔️ | ✔️ | ✔️ | ✔️ |  |  |  |
| Peak et al 2010 | ✔️ | ✔️ | ✔️ | ✔️ | ✔️ |  | ✔️ |  |  |  |
| Radina et al 2011 | ✔️ | ✔️ | ✔️ | ✔️ | ✔️ | ✔️ | ✔️ |  | ✔️ | ✔️ |
| Ragusa & Crampton 2019 | ✔️ | ✔️ | ✔️ | ✔️ | ✔️ |  | ✔️ |  |  |  |
| Ramirez et al 2015 | ✔️ | ✔️ | ✔️ | ✔️ |  | ✔️ | ✔️ |  |  |  |
| Ray et al 2017 | ✔️ | ✔️ | ✔️ |  | ✔️ |  | ✔️ |  |  |  |
| Reghagwa & Ono 2021 | ✔️ | ✔️ | ✔️ | ✔️ | ✔️ | ✔️ | ✔️ | ✔️ |  | ✔️ |
| Ren et al 2019 | ✔️ | ✔️ | ✔️ | ✔️ | ✔️ | ✔️ | ✔️ |  |  |  |
| Renahy et al 2010 | ✔️ | ✔️ | ✔️ | ✔️ | ✔️ |  | ✔️ |  |  |  |
| Rooks et al 2012 | ✔️ | ✔️ | ✔️ | ✔️ | ✔️ |  | ✔️ |  |  |  |
| Rose & Friedman 2013 | ✔️ | ✔️ |  |  | ✔️ |  | ✔️ |  |  | ✔️ |
| Rust & Davis 2011 | ✔️ | ✔️ | ✔️ |  | ✔️ |  | ✔️ |  |  | ✔️ |
| Scantlebury et al 2017 | ✔️ | ✔️ | ✔️ | ✔️ | ✔️ | ✔️ | ✔️ |  |  | ✔️ |
| Schladen et al 2011 | ✔️ | ✔️ |  |  | ✔️ |  | ✔️ | ✔️ |  |  |
| Schmidt et al 2021 | ✔️ | ✔️ |  | ✔️ | ✔️ |  | ✔️ |  |  |  |
| Schrank et al 2010 | ✔️ | ✔️ | ✔️ | ✔️ |  | ✔️ | ✔️ |  | ✔️ | ✔️ |
| Seckin 2014 | ✔️ | ✔️ | ✔️ | ✔️ | ✔️ |  | ✔️ |  |  |  |
| Seckin 2020 | ✔️ | ✔️ | ✔️ | ✔️ | ✔️ |  | ✔️ |  |  |  |
| Shum et al 2014 | ✔️ | ✔️ | ✔️ |  | ✔️ |  | ✔️ |  |  | ✔️ |
| Smith et al 2015 | ✔️ | ✔️ | ✔️ | ✔️ | ✔️ |  | ✔️ |  |  | ✔️ |
| Soni et al 2017 | ✔️ | ✔️ | ✔️ | ✔️ | ✔️ | ✔️ | ✔️ |  |  |  |
| Suri et al 2016 | ✔️ | ✔️ | ✔️ | ✔️ | ✔️ | ✔️ | ✔️ |  |  |  |
| Tan & Goonawardene 2017 | ✔️ | ✔️ | ✔️ |  |  |  | ✔️ |  | ✔️ |  |
| Terry et al 2016 | ✔️ | ✔️ | ✔️ | ✔️ | ✔️ |  | ✔️ |  |  |  |
| Turnbull et al 2021 | ✔️ | ✔️ | ✔️ | ✔️ | ✔️ | ✔️ | ✔️ | ✔️ |  |  |
| Vader et al 2011 | ✔️ | ✔️ | ✔️ | ✔️ | ✔️ |  | ✔️ |  |  |  |
| Vamos et al 2019 | ✔️ | ✔️ | ✔️ | ✔️ | ✔️ | ✔️ | ✔️ |  |  | ✔️ |
| Viswanath et al 2013 | ✔️ | ✔️ | ✔️ | ✔️ | ✔️ | ✔️ | ✔️ |  |  |  |
| Waldman et al 2018 | ✔️ | ✔️ | ✔️ | ✔️ | ✔️ | ✔️ | ✔️ | ✔️ |  |  |
| Wang et al 2022 | ✔️ | ✔️ | ✔️ | ✔️ | ✔️ |  | ✔️ |  |  |  |
| Weber et al 2020 | ✔️ | ✔️ | ✔️ | ✔️ | ✔️ |  | ✔️ |  |  |  |
| Wlodarczyk et al 2019 | ✔️ | ✔️ | ✔️ | ✔️ | ✔️ |  | ✔️ |  |  |  |
| Yamashita et al 2019 | ✔️ | ✔️ | ✔️ | ✔️ | ✔️ |  | ✔️ |  |  |  |
| Yi 2015a | ✔️ | ✔️ | ✔️ |  | ✔️ |  | ✔️ | ✔️ |  | ✔️ |
| Yi 2015b | ✔️ | ✔️ | ✔️ | ✔️ | ✔️ |  | ✔️ | ✔️ |  | ✔️ |
| Yi et al 2012 | ✔️ | ✔️ | ✔️ | ✔️ | ✔️ | ✔️ |  | ✔️ |  |  |
| Yoon et al 2017 | ✔️ | ✔️ |  | ✔️ | ✔️ |  | ✔ |  |  | ✔️ |
| Yusup et al 2019 | ✔️ | ✔️ | ✔️ | ✔️ | ✔️ | ✔️ | ✔️ |  |  |  |
| Zach et al 2011 | ✔️ | ✔️ | ✔️ | ✔️ | ✔️ | ✔️ | ✔️ |  |  | ✔️ |
| Zaim et al 2021 | ✔️ | ✔️ | ✔️ | ✔️ | ✔️ |  | ✔️ |  |  |  |
| Zhang et al 2020 | ✔️ | ✔️ | ✔️ |  | ✔️ |  | ✔️ | ✔️ |  |  |
| Zhao et al 2021 | ✔️ | ✔️ | ✔️ | ✔️ | ✔️ |  | ✔️ |  |  |  |
| Zulman et al 2013 | ✔️ | ✔️ | ✔️ | ✔️ | ✔️ | ✔️ | ✔️ |  |  |  |
